# Requirements and Barriers for Human-Centered SMEs

**DOI:** 10.3390/s24144681

**Published:** 2024-07-19

**Authors:** Julia Nazarejova, Zuzana Soltysova, Tetiana Rudeichuk

**Affiliations:** Faculty of Manufacturing Technologies, Technical University of Kosice, 080 01 Presov, Slovakia; zuzana.soltysova@tuke.sk

**Keywords:** Industry 5.0, SMEs, human-centered, sustainability, resilience, digital transformation, sensors

## Abstract

With the advantages of new technologies and rising demand from customers, it is necessary to improve the manufacturing process. This necessity was recognized by the industry; therefore, the concept of Industry 4.0 has been implemented in various areas of manufacturing and services. The backbone and main aspect of Industry 4.0 is digitalization and the implementation of technologies into processes. While this concept helps manufacturers with the modernization and optimization of many attributes of the processes, Industry 5.0 takes a step further and brings importance to the human factor of industry practice, together with sustainability and resilience. The concept of Industry 5.0 contributes to the idea of creating a sustainable, prosperous, and human-friendly environment within companies. The main focus of the article is to analyze the existing literature regarding what is missing from the successful implementation of human centricity into industry practice, namely in small and medium-sized factories (SMEs). These findings are then presented in the form of requirements and barriers for the implementation of human centricity into SME factories, which can serve as guidelines for implementing human-centered manufacturing using axiomatic design theory in SMEs, which can serve as a roadmap for practitioners.

## 1. Introduction

The concept of industrial revolution (IR) is widely known to most people. This phenomenon is generally associated with changes in the manufacturing environment but also has an impact on social, political, and other aspects of people’s lives [1,2]. Regarding these industrial revolutions, each revolution is significant in its own way. Generally, these revolutions are recognized by the characteristic contributions they brought to the industrial world during their period [3]. Mechanization, waterpower, and steam power are representatives of the first industrial revolution known as Industry 1.0. The second industrial revolution was related to mass production and finding the most efficient production system, which was famously addressed by Henry Ford’s assembly line [4]. In the late 20th century, digitalization and the innovation of personal computers once again changed the face of the industry in the form of the third industrial revolution (IR 3.0). The concept of the fourth industrial revolution, introduced by the German government in 2011, has brought terms such as the internet of things, big data, cyber-physical systems, smart factories, smart sensors, and digital twins into the industry’s vocabulary [5]. When these remarkable ideas from that era are closely examined, there is a common relation among them—it is the effort to bring higher productivity and greater profit. As it is well-known, every innovative idea can bring great advantages but also may cause problems [6].

With technological advancements not only in manufacturing but also in healthcare, populations on most continents have started to age and birth rates have decreased [7]. This is where the shortcomings of industrial revolutions become evident. The innovations introduced for increased productivity do not take into consideration the human worker as an important factor in the manufacturing system [8]. Due to this reason, Industrial Revolution 5.0 comes in.

Some literature recognizes the fifth industrial revolution as a prolongation of Industry 4.0. The concept of this movement is based on three main pillars: sustainability, resilience, and human centricity [9,10,11]. These objectives are recognized as crucial points for the stability and prosperity of factories and society [12]. Industry 4.0 emphasizes the importance of human-centric approaches by promoting a new mindset of curiosity and openness toward new technologies throughout the enterprise [13,14]. Previous authors [15] have described the possible consequences of digitalization in relation to new technologies. The implementation of digital transformation poses particular challenges for SMEs [16]. The importance of digital capabilities, structured communication, and self-learning information processing are highlighted for businesses in the context of Industry 4.0. Understanding and implementing the principles and capabilities outlined in a previous paper [17] can potentially pave the way for future advancements toward Industry 5.0, which is expected to focus on human–machine collaboration, decentralized decision-making, and a more sustainable approach to manufacturing. While the fourth IR is characterized by its technological advancements and focus on automation and efficiency, Industry 5.0 emphasizes the collaboration between humans and machines, aiming for personalization, sustainability, and the enhancement of human roles in the production environment. This evolution marks a shift from purely technology-driven processes to more human-centered and ethical manufacturing practices. In relation to Industry 4.0, Scoop [18] discussed in his work the technological advancements and their impact on manufacturing efficiency and automation. On the other side, Industry 5.0 explores the shift towards human-centered production, emphasizing collaboration between humans and machines for more personalized and sustainable manufacturing [19]. Industry 5.0 redefines manufacturing by integrating advanced technologies in a way that enhances human roles, prioritizes sustainability, and adheres to ethical standards. It moves beyond the automation focus of Industry 4.0 to create a collaborative, innovative, and human-centric manufacturing environment. This paradigm shift aims to harness the best of both human ingenuity and technological advancements, leading to more sustainable and fulfilling manufacturing practices. Based on that, one can state that IR 5.0 tries to address problems that were created with the implementation of other IRs. Pollution, the excessive use of environmental resources, unpreparedness for crisis situations such as pandemics and economic crises, and a lack of human labor are just a few examples of why a new perspective on the industry is so important [8,12,20]. Even if some companies may not feel the effects of these problems yet, it is more than likely that they are going to face them in the near future. 

The main goal of a human-centric industry is to recognize people’s intelligence, talent, and subjectivity and unite them with more precise and effective artificial intelligence (AI) [21]. Additionally, there is a necessity for great emphasis on improving human well-being, needs, and rights. There can be similar attempts seen to bring elements of sustainability and resilience in previous IRs, but from the perspective of prior IR concepts, human-centered (HC) manufacturing is a novelty [12,20]. 

As was stated in the previous paragraph, human-centered manufacturing prioritizes the wellness, needs, and prosperity of workers and the designing of manufacturing processes is subordinate to these terms. Therefore, there is a need for supportive tools that can help enhance this transformation in design and, subsequently, the usage of this concept. One answer for this could be sensors, which have great potential not just in IR 5.0 but also in human-centered aspects. Sensors could be used in the various elements of manufacturing processes. To illustrate their potential, we present the following examples of the usage of sensor technologies in the HC industry:

- Sensors for safety monitoring: Safety is and always should be the main priority of every employer, not just in the human-centered concept. Therefore, sensors that provide information on working conditions like the presence of toxic gases or excessive noise levels are very much needed. In terms of monitoring the well-being of the worker, wearable sensors can provide data about the health and emotional condition of the worker.
- Sensors for ergonomic and monitoring of worker well-being. Ergonomics questions are prominent in various work positions. Sensors could help with indicating the incorrect movement and posture of the worker and help prevent the risk of injuries or musculoskeletal disorders. Also, environmental conditions could be monitored by sensors in terms of air and humidity conditions or lighting intensity.
- Sensors for monitoring efficiency and productivity: To ensure efficiency and productivity, sensors provide real-time information that can help workers reduce errors and maintain production plans.
- Sensors for monitoring human–robot collaboration: To ensure successful collaboration between humans and robots, sensors could play a role as a safety component in preventing collision and accidents between both parties. In terms of adaptation, sensors could support adaptation based on human worker actions.
- Sensors to enhance data-driven decision making: Decision making is crucial to ensuring the prosperity and competitiveness of companies, therefore data and real-time information are important to gain insight into workers’ performance. This information can be used for strategic planning, operational planning, and resource planning.

As can be seen in the examples provided of sensor usage, the application of sensors is crucial for the smooth implementation of human centricity in factories.

According to the World Bank, small and medium-sized enterprises (SMEs) represent approximately 90% of businesses and more than 50% of employment worldwide. Small and medium-sized enterprises are mostly described by the number of employees [22]. The number of employees in a small enterprise is 10 to 49, and for medium-sized enterprises, it is between 50 and 249 employees. Despite their prominent representation in numbers, SMEs are more prone to problems [9]. For that reason, it is important to help SMEs recognize the opportunities that Industry 5.0 brings to them and help them determine how to survive and thrive.

At the beginning of this study, it is possible to say that Industry 5.0 is a relatively new concept and not so completely developed, especially in terms of SMEs.

The main research questions that this publication can help find answers to or at least shed more light on are as follows:

- What are the requirements for SMEs to implement Industry 5.0?
- What are the barriers for SMEs to implement Industry 5.0?

## 2. Materials and Methods

**Figure 1:**
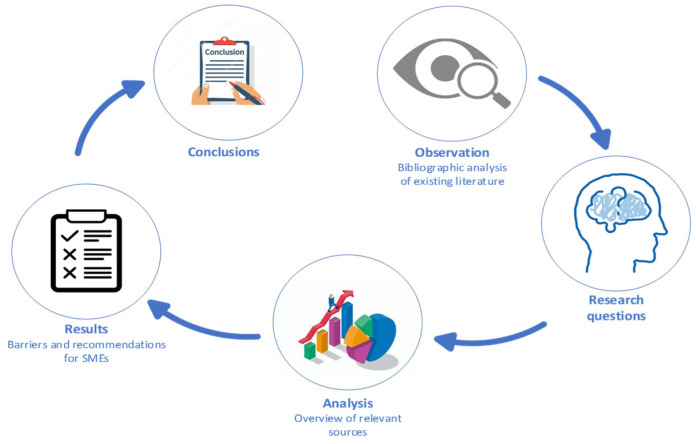
Methodology mapping in the paper.

The basis of this paper is to provide a bibliometric analysis to identify the main barriers and recommendations for SMEs in terms of human-centered manufacturing. A comprehensive delineation of the research methodology used in this paper is supported by the illustration in Figure 1. 

Following this methodology, this study is divided into 5 main parts. In the first part, the existing literature is subjected to bibliographic analysis using the Scopus database, in which the search terms were “human-centered” and “SMEs” and “Industry 5.0”, searching within all fields up to 14 April 2024. Together, there were 173 publications found in the Scopus database. Then, this literature was analyzed and categorized based on the number of publications per year, the main research discipline, the country of publication, and the keyword co-occurrence with the most used keywords in this domain. To find answers to the research questions specified in the Introduction section, it was necessary to decompose the existing literature. Subsequently, an overview of the relevant research is provided. After that, the main requirements and barriers for human-centered factories are presented using Axiomatic design theory extracted from the overview of relevant sources. Lastly, the final findings and answers to research questions are summarized in the conclusion section. 

## 3. Bibliometric Analysis and Literature Review of Relevant Publications

The first step of bibliometric analysis [23] will be used to provide an introduction to the study on the proposed aim of the paper in terms of the interest evaluation of this topic. The next part of this section is dedicated to reviewing relevant publications. 

### 3.1. Evaluation of Literature Sources by Bibliometric Analysis Methods

The bibliometric analysis was focused on implementing Industry 5.0 in SMEs in relation to human-centered manufacturing using the Scopus database. The Scopus database was queried based on the following terms: “human-centered” and “SMEs” and “Industry 5.0”, searching within all fields. Even though this topic of research is new, there is a relatively large amount of publications on this subject. To date, as of 14 April 2024, there are 173 publications in the Scopus database. The bibliographic analysis was provided from the perspective of the number of publications per year, the research areas of publication, and the countries and territories of publication, and a word map was created from the occurrence of keywords in these publications. 

**Figure 2:**
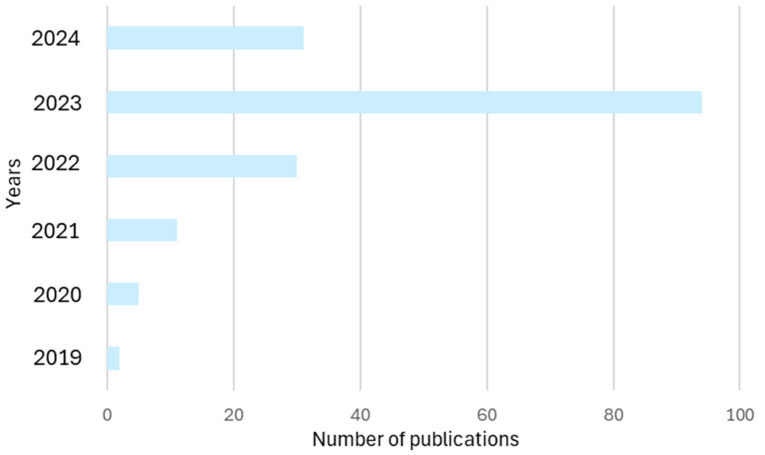
The number of publications per year.

The number of publications per year between 2019 and 2024 is presented in Figure 2. One can see that during the years 2019 to 2023, the number of publications showed an increasing trend. The highest number of publications occurred in 2023, with precisely 94. Even though there are only 31 publications in 2024, it can be predicted that this trend will continue in 2024 as this field is relatively popular in research and manufacturing areas. 

**Figure 3:**
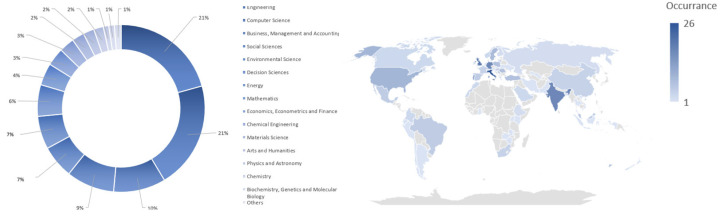
The percentage occurrence of publications in different research disciplines (on the **left**) and the publication occurrence based on country of origin (on the **right**).

This research topic is represented in a wide range of areas, as can be seen in Figure 3, which illustrates the percentage occurrence of publications in different research disciplines and the publication occurrence based on country of origin.

As can be seen, the most relevant areas are Engineering (21%), Computer Science (21%), and Business, management, and accounting (10%). Looking at the origin country of publications, the highest publication occurrence is in Italy (26 publications), and from the Asian continent, India has the greatest number of publications, with precisely 17.

**Figure 4:**
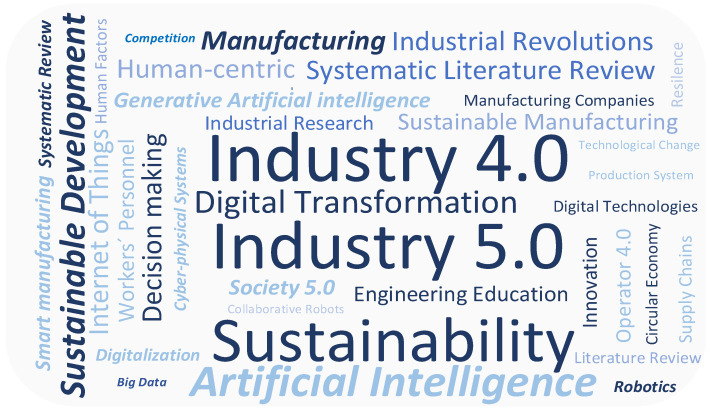
Keyword occurrence within the publications about human-centered factories.

Following this, a word map was created from the most cited keywords occurring in publications about human-centered manufacturing. The keyword frequency in this word map is indicated by the font size. To create this word map, only keywords that have a frequency of occurrence of 5 or more were used (see Figure 4).

As can be seen from this figure, the most frequently occurring keywords are Industry 4.0 (76 times) and Industry 5.0 (58 times). This fact indicates, as mentioned in the Introduction section, that IR5 is a prologue to IR4, and due to this reason, they are closely related. The remaining significant keywords are sustainability, artificial Intelligence, sustainable development, manufacturing, etc. 

When analyzing the most-used keywords, “Industry 5.0” represents the integration of human creativity and skills with the precision and efficiency of advanced technologies, with the aim of creating a more sustainable, efficient, and human-centric manufacturing environment. In relation to this, sensors play a crucial role in this transition by enabling the seamless interaction between humans and machines, enhancing safety, efficiency, and personalization. This is how sensors are connected to human-centered manufacturing in Industry 5.0. To look at this connection more closely, when analyzing the literature, there is no review paper focusing on sensors used for human-centered manufacturing in general. Due to this reason, the next sub-section will be focused on the literature analysis from diverse perspectives of sensor applications in human-centered manufacturing. 

### 3.2. Analysis of Sensors Applications in Human-Centered Manufacturing

As mentioned in the Introduction section, sensors are interconnected in the domains of safety, ergonomics and worker well-being, efficiency and productivity, human–robot collaboration, and data-driven decision making. For this purpose, the most relatable literature sources are analyzed and categorized from these perspectives, as can be seen in Table 1.

Based on the sensors used in human-centered manufacturing analysis, it can be stated that these articles are relatively recent, as they were published between 2021 and 2024. Most of them are oriented toward sensors in human-centered manufacturing with a focus on safety, human–robot collaboration, ergonomics, and worker well-being, which confirms the connection/relationship between human-centered manufacturing, Industry 5.0, and fundamental smart technology regarding sensors as they are considered essential components for smart systems to perceive and interact with their environment. 

### 3.3. Requirements and Barriers for Human-Centered SME Factories—Overview of Relevant Sources

The most relevant literature sources in relation to the specified fields are selected, where the main aim is described together with the requirements and barriers identified for human-centered SME factories, while additional information is added—the number of citations per document (see Table 2). 

Based on the primary literature source, it is safe to say that the application of human centricity has great potential in various industry sectors and society as a whole. In the next section, some of the barriers and requirements are listed and summarized. Industry 5.0 is still a relatively new paradigm and, therefore, even publications and studies are in the initial state of matter [47]. In addition, there is a need for a multidisciplinary point of view regarding issues of HC practice, such as ergonomics, psychology, aesthetics, economics, and medicine, in addition to more traditional disciplines [46]. 

Firstly, based on the study, one can state that most of the requirements are also barriers to the implementation of HC into industrial practice. For example, there is a call for new technologies that are compatible with working with humans, which can be taken as a requirement but also as a barrier because these technologies need time to be tested [4,36]. There is also a need for skilled workers that can be implemented into the HC process, but in the market, there is a lack of skilled and knowledgeable potential workers. With regard to potential employees, not everyone is inclined to changes regarding cooperation with robots and learning new skills. This can depend on personal traits, the age of the worker, and other factors [45]. Collaboration between robots and humans also brings its own nuances, such as regulatory, social, ethical, psychological, and aesthetic issues and other crucial aspects [4,37,38]. 

In terms of determining how to implement such considerable and important changes, there are still not enough studies and outlines to ensure a smooth and secure transformation [12,45,48]. Another critical point is determining how to conduct measurements and what indicators to apply to evaluate the success of HC performance in the processes. Another possible way to tackle this research gap is the utilization of academic learning factories based on specific areas of institutions. There is also pressure regarding the time required for the development of new practices to implement human-centered manufacturing approaches [35,43]. 

Based on the studies and experiences, one could state that the implementation of Industry 4.0 into manufacturing and service processes bore fruit but also put workers in a difficult position. The current maturity models for Industry 4.0 are inadequate for HC, which can hinder the transformation to Industry 5.0 [39]. Most of the concepts in production technologies are based on uniformity, which can be problematic with the implementation of HC, as the HC concept highlights the individual quality and uniqueness of each and every person. Regarding these aspects, it is challenging to ensure transformation from a fully technological perspective to a balanced human-centric perspective. 

In the matter of questions about security, significant improvements are required to avoid cyberattacks, as digital evolution in the Society 5.0 concept is closely linked to IoT technologies, which can be vulnerable to hackers. 

Lastly, the HC aspects are usually presented as additional features, in contrast to taking HC as an integral part of the technology development process, which can bring prosperity to companies and society. The study, implementation, and development of new strategies, measurements, and tools in terms of such a broad concept as Industry 5.0 and, specifically, HC require time, knowledge, and, last but not least, funding. The necessity to emphasize the importance of Industry 5.0 should also be a priority for governments, in terms of the prosperity of the countries, as well as the health and contentment of people working in the industry. 

**Figure 5:**
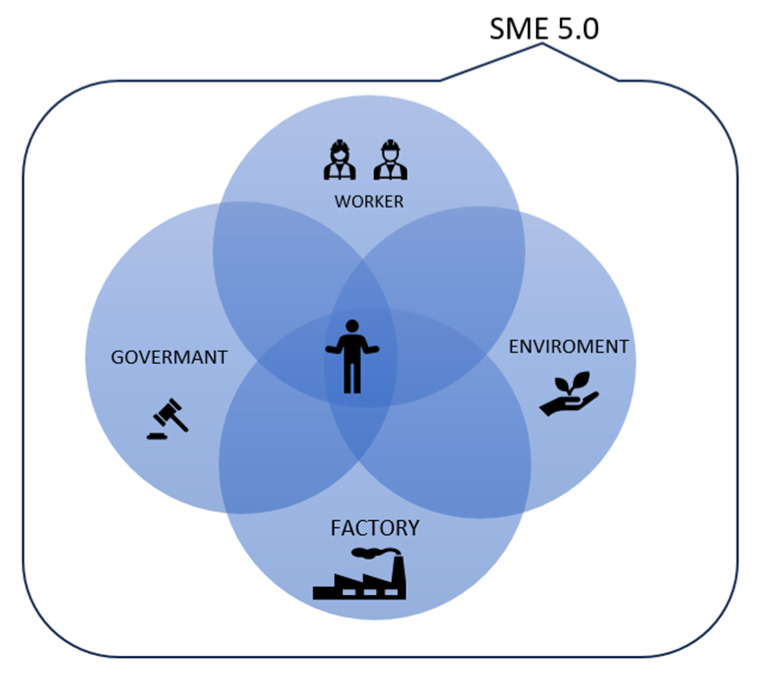
Keyword occurrence within the publications.

This brings us to the conclusion that all aspects of Industry 5.0 (sustainability, human-centricity, and resilience) are tightly connected. One can state that the application of Industry 4.0 technologies and HC, together with sustainability values, can improve the resilience and overall prosperity of companies and the quality of life and the environment. According to these notes about the barriers and requirements for human-centered SMEs from the existing literature, the human-centered manufacturing system can be analyzed from four perspectives impacting humans—the worker perspective, the factory perspective, the government perspective, and the environment perspective (see Figure 5).

Then, the requirements and barriers for human-centered factories can be analyzed and the recommendations will be provided in the next section in terms of the worker perspective and the factory perspective.

## 4. Presentation of Recommendations for Human-Centered Factories Using Axiomatic Design Analysis

The recommendations for factories will be presented here using Axiomatic design (AD)-based theory, as this theory is rarely associated with this issue. The theory of Axiomatic design is generally dedicated to the system design methodology, which uses design matrices to systematically analyze the transformation of customer needs (CNs) into functional requirements (FRs), design parameters (DPs), and process variables (PVs) [32]. Here, some important works dedicated to AD theory can be mentioned, such as [49,50,51]. DPs will be represented as recommendations for human-centered SMEs regarding how to satisfy the specified FRs in the form of requirements presented from two perspectives selected from the four we defined—the worker and factory perspectives. The relationship between a FR and a DP is represented by ‘X’ in mentioned matrix. These recommendations can be used as guidelines for SMEs regarding what to apply/what is needed to be a human-centered factory. From the provided literature analysis, the main barriers were selected and then categorized based on the two perspectives (worker and factory) with a proposition of recommendations. The last sub-section of this section will summarize the main contributions of this article. 

### 4.1. Worker Perspective Base Analysis

This subsection is dedicated to the worker perspective regarding the attributes of humans in the manufacturing process. The analysis is based on the AD theory. The highest levels of FRs and DPs, with their sub-levels from the physical, intellectual, and emotional points of view, are shown in the following figures.

#### 4.1.1. Physical View 

**Figure 6:**
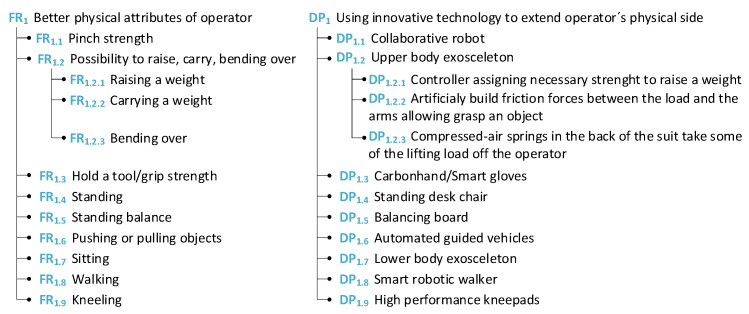
Physical attributes of operators.

In this subsection of the study, the physical attributes of the worker are addressed and some suggestions are presented. These attributes are one of the most studied and addressed in the literature and in practice because, for a long time, physical discomfort was the most common factor for work leave (see Figure 6).

**Figure 7:**
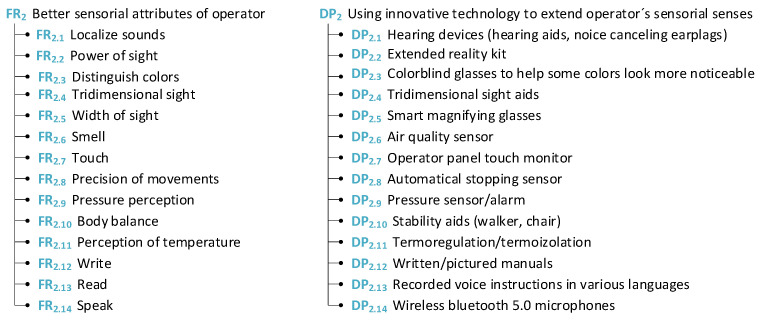
Sensorial attributes of operators.

Regarding the physical viewpoint from the worker’s perspective, it is important for them to have better physical attributes in diverse areas of their work, which are key elements for a company to achieve. For example, a company would like to raise worker standards, which means producing more products in less time. This impacts the physical health of the worker, such as increased walking and lifting heavier weights. In this case, the company should ensure the provision of upper-body exoskeletons to facilitate these aspects in order to meet higher work standards. These guidelines present the main barriers from the worker’s perspective, summarized from existing literature sources, and propose solutions for companies to avoid these obstacles (see Figure 7).

**Figure 8:**
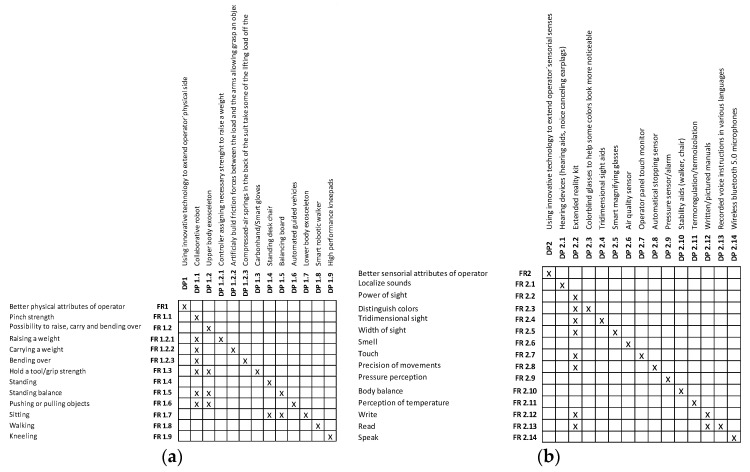
Matrices (**a**) for physical attributes and (**b**) sensorial attributes of operators.

It is important to note that the method used is based on the AD theory to provide a so-called manual, and not a study design based on the usage of axioms. The structures are then supported and clarified by matrices for Figure 8a physical attributes and Figure 8b sensorial attributes of the operator, which provide insight into the link between the DPs and FRs from a physical perspective (see Figure 8). These matrices could be taken into account for decision making in the question of how to improve the physical and sensorial attributes of workers.

As can be seen in the matrices, some of the suggested improvements can be used to solve more than one of the presented attributes. For instance, collaborative robots can help with at least seven of the presented attributes. 

#### 4.1.2. Worker Intellectual View

**Figure 9:**
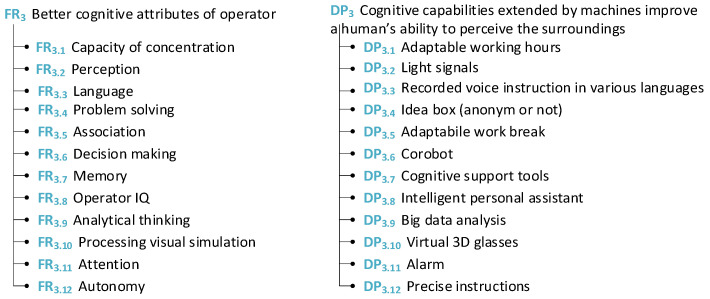
Cognitive attributes of operators.

The following section is dedicated to the exploration of the worker from an intellectual point of view. This group is divided according to the possibilities of how to enhance the cognitive attributes and skill set of the worker (see Figure 9). 

Under the label of an intellectual perspective, attributes such as language, memory, and IQ were put into the group of cognitive attributes. 

**Figure 10:**
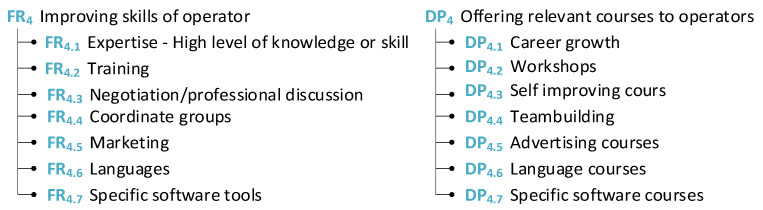
Skills of operators.

Afterward, AD theory was used to provide ideas to improve the skill sets of workers. There are some examples provided of what could be used to improve the skill sets of workers and what tools and methods could be used for this purpose (see Figure 10).

**Figure 11:**
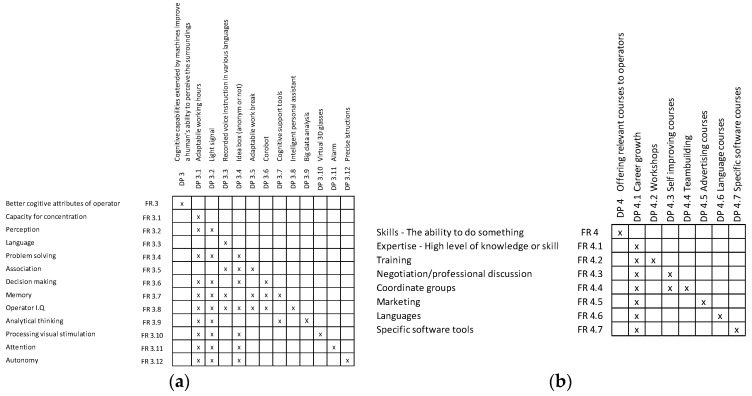
Matrices of (**a**) cognitive attributes of operators and (**b**) skills of operators.

Similarly for this group of attributes matrices, Figure 11a the cognitive attributes of operators and Figure 11b skills of operator were created. For example, cover growth can be achieved by developing skills and acquiring useful knowledge. The possibility of achieving a higher position in the company can be motivation for workers, for example, attendance to courses or obtaining higher education. 

#### 4.1.3. Emotional View 

**Figure 12:**
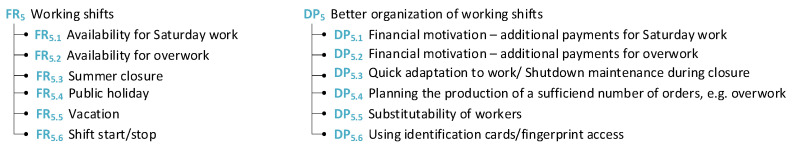
Personal needs related to private life.

To create a more human-centered workplace, the emotional aspect of human beings should also be taken into consideration. For this purpose, two factions were investigated. In the first group, we assigned attributes connected to personal needs and private life; furthermore, the second group is dedicated to workers’ emotional responses and behavior (see Figure 12). 

**Figure 13:**
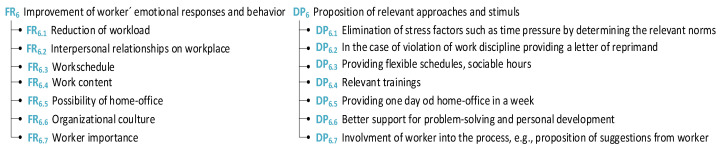
Worker’s emotional responses and behavior.

In terms of working shifts, this topic became more prominent due to the effect of COVID-19, in which many employees were partially working from their home office. In many professions, this even became the preferable way of working. Regarding the availability for overwork/overtime, financial motivation or additional leave seems to be the strongest motivation (see Figure 13). 

The last study group of attributes from a manufacturing point of view is related to the emotional responses of the worker. The solution should contribute to bringing more contentedness to the emotional state of the worker, in terms of self-importance, good relationships between co-workers/management and employees, etc. 

**Figure 14:**
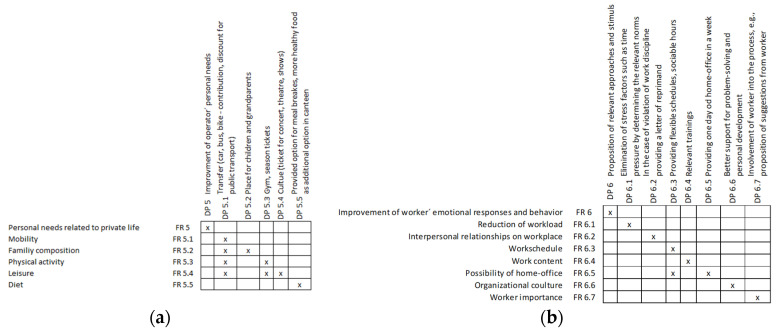
Matrices of (**a**) personal needs related to private life and (**b**) worker’s emotional responses and behavior.

For both groups of attributes presented above, the matrices of Figure 14a personal needs related to private life and Figure 14b worker’s emotional responses and behavior are also provided (see Figure 14). 

### 4.2. Factory Perspective Based Analyze

**Figure 15:**
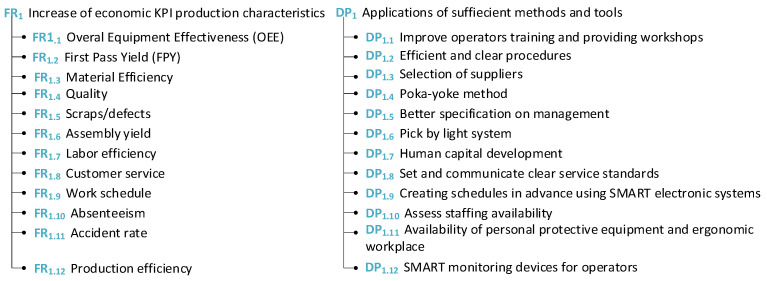
Economic KPI production characteristics.

**Figure 16:**
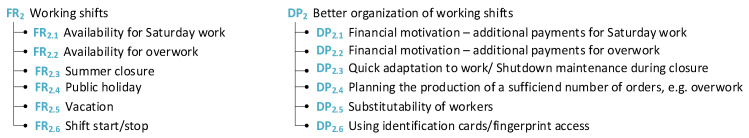
Worker shifts—factory view.

**Figure 17:**
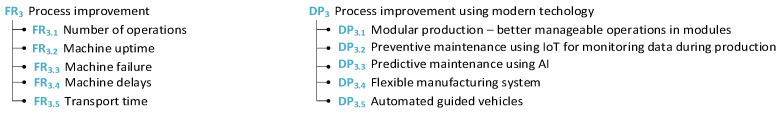
Process improvement.

This section provides insight into the factory perspective and analyses are carried out using the AD theory, similar to the worker perspective. Firstly, economic KPI production characteristics are implemented into the AD-based method and matrices are provided (see Figure 15, Figure 16 and Figure 17). 

These barriers summarized from the company perspective can be satisfied by the proposed solutions in the form of design parameters. For problems such as workers not being available due to vacations or illness and prolonged absences from work, the best solution for the company in this case is to consider worker substitutability. These guidelines can be helpful for companies as they can determine what the main problem is and what the proposed solution is.

**Figure 18:**
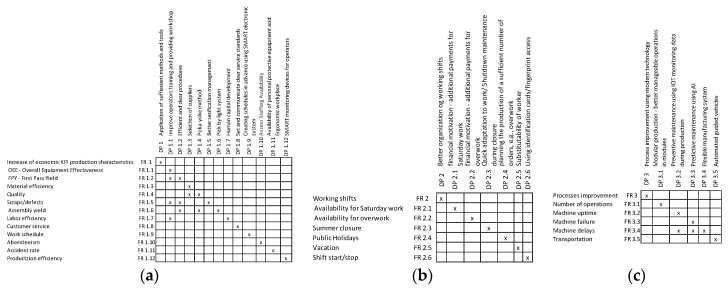
Matrices of (**a**) economic KPI production characteristics, (**b**) worker shifts—factory view, and (**c**) process improvement.

To clearly identify the link between the DPs and FRs, the following matrices for each high-level FR_1–3_–DP_1–3_ have been created. Namely, matrices for Figure 18a economic KPI production characteristics, Figure 18b workers shifts—factory view, and Figure 18c process improvement can be seen in Figure 18. 

The last matrices also provide suggestions for how to implement suggestions for factories in terms of human centricity. In terms of work shifts, similar to the worker perspective, we include process, customer, quality, and material attributes. 

A list of all the abbreviations can be found in Table 3.

The next section discusses the requirements and barriers for human-centered factories in terms of the government perspective and the environmental perspective.

## 5. Human-Centered Factories in Terms of Government and Environment Perspective

Regarding the environmental aspect of the study, the average time spent in the workplace is eight and a half hours; this time should be spent in a suitable working environment in terms of air quality, waste regulation, etc. On the other hand, manufacturing and factories, with their actions, also affect the environment, even outside their gates. In this regard, there are already various movements and designs that could be adjusted to be valid for HC implementation. Furthermore, for government attributes, it is a positive sign that motions such as Industry 5.0 are put into motion by the European Commission. However, additional funding is also important to carry out more studies and tests and to bring more attention to human-centricity in the industry and, more specifically, its application in SMEs. The government could support companies that are practicing HC or are interested in implementing some of the HC ideas; for instance, in the form of reduced tax credits, establishing a fund for HC applications, and helping to increase awareness about this topic. There is also a need for further education on this topic at multiple levels of the educational system and in the manufacturing sector. One could state that it should be in the interest of the government itself to help people feel better and be successful in their working positions, for instance, to reduce unemployment and work fluctuation, which can be seen as modern-day problems in the labor market.

## 6. Contributions

The analysis of the existing literature highlights the critical requirements and barriers faced by human-centered SMEs. The integration of advanced technologies, employee well-being, customer-centric innovation, and organizational agility are key requirements. However, resource constraints, resistance to change, and regulatory issues pose significant barriers. Strategies such as collaborative networks, government support, and tailored training programs can help SMEs navigate these challenges and achieve a human-centered approach to business operations. These insights provide a comprehensive understanding of the evolving needs and obstacles in the context of human-centered SMEs.

The main contribution resulting from this review paper can be seen as the development of a framework in the form of guidelines for better integration of human-centered principles and AD, providing practical examples to incorporate humans into production more smoothly for HC SMEs. In human-centered manufacturing, the theory of axiomatic design suggests that a single design parameter can be effectively satisfied by multiple requirements. This principle highlights the importance of designing systems that can address diverse user/worker needs and operational requirements through integrated and cohesive design choices. By strategically selecting and implementing design parameters that cater to various aspects of usability, ergonomics, efficiency, and user/worker satisfaction, manufacturers can create products and processes that enhance the overall human experience and optimize operational performance. This approach not only simplifies the design process but also promotes holistic solutions that prioritize the well-being and productivity of users/workers within manufacturing environments. This contribution can be seen in the offering of empirical evidence that supports the effectiveness of combining human-centered approaches with AD principles in improving productivity, quality, and employee well-being in SMEs, while SMEs can arbitrarily adopt this framework.

In terms of the environment, we propose that workers are affected by the company’s procedures not just during working shifts but also outside the factory gates.

We also provided a literature review regarding the application of sensors in HC manufacturing, which, to our knowledge, has not been performed before. The main aim of this overview contributes via the provision of insights into how sensor technologies enhance manufacturing processes to prioritize human factors such as safety, efficiency, and ergonomic design. Sensors can monitor environmental conditions, detect human presence, measure physical parameters, and enable real-time feedback systems, all of which collectively improve the working conditions and productivity of human operators. In relation to the article’s contribution, the literature analysis reveals potential gaps in the existing literature on sensors in human-centered manufacturing systems, which can be addressed in future investigations that can contribute to safer, more efficient, and adaptive manufacturing environments:

- In relation to integration challenges, despite advancements, integrating diverse sensors into existing production systems may still pose challenges related to compatibility, data interoperability, and cybersecurity.
- In relation to scalability, many existing studies are focused on pilot implementations or specific cases. There could be a gap in the understanding of the scalability of sensor-based solutions across various industries, production scales, and regulatory environments.
- In relation to human factors, it is known that such sensors offer benefits on the one hand, but on the other hand, their deployment should consider human factors, i.e., worker privacy concerns, the acceptance of sensor technologies, and the potential impacts on job satisfaction and workload.
- In relation to costs, there is a need for more studies examining the cost-effectiveness and return on investment of sensor applications in an HC environment. Evidence of economic benefits could accelerate the adoption of these sensors in practice.
- In relation to ethical and legal issues, as sensor data collection expands, there is a gap in addressing ethical considerations such as data privacy and legal issues such as regulatory compliance surrounding sensor use in an HC environment.

## 7. Conclusions

The concept of a human-centered factory is a very broad and interdisciplinary theme, as various kinds of aspects are involved in this approach. However, from our literature review, it can be concluded that the most influential factors in this context are as follows: the worker, the factory/employer, the government, and environmental aspects. To address the research questions stated earlier in this study, one can assert that the requirements and barriers are tightly connected. Section 4 is devoted to this issue and answers these research questions. For example, it is necessary to have skilled workers, but in the market, there are not enough skilled people.

To outline the findings from the existing literature, an analysis of the literature sources was provided by bibliometric analysis and a deeper analysis of selected literature publications:

- Based on our literature review, the two most prominent aspects were analyzed, and suggestions in the form of the AD theory were implemented for human-centered factories. In this manuscript, all aspects were focused on the barriers and recommendations from the worker and factory/employer perspectives. However, it is necessary to also mention the government and environmental aspects, which were discussed in Section 5.
- Information plays a crucial role in every aspect of the manufacturing process, particularly in the implementation of new paradigms like HC manufacturing. To ensure quick and effective decision-making, the right kinds of data, and of the required quality, are needed. Sensors are implemented into manufacturing processes to obtain data and information. Section 3.2 is dedicated to the literature review of sensor implementation in HC manufacturing. In summary, based on the literature, the potential research gaps were presented as the connection between HC manufacturing and sensors plays a crucial role in enhancing safety, efficiency, and ergonomics in human-centered factories.
  These implications/recommendations could be used not only in SMEs but also in larger companies, with bigger or smaller modifications, in terms of the usability, the financial state of the company, etc.

To offer more valuable insights for practitioners and researchers seeking to apply these concepts in real-world settings, the company Toyota has long been a pioneer in HC manufacturing for its adoption of human-centered production methods, such as integrating worker feedback into production line adjustments, which has significantly enhanced both efficiency and worker satisfaction. Similarly, the Bosch company has implemented human–robot collaboration systems that prioritize worker safety and ergonomic support, leading to a marked increase in productivity and a decrease in workplace injuries. As another example, the well-known company BMW has adopted HC production in its production lines by introducing exoskeletons for workers. These wearable devices help reduce physical strain and improve overall worker well-being. The implementation of exoskeletons has resulted in lower injury rates and higher productivity. The integration of advanced sensors and robotics to assist human workers is a key element of the human-centered strategy and represents a successful blend of technology and human factors, which can be considered the main aim of the Industry 5.0 concept.

Regarding future work, the axiomatic design theory could be applied to the government and environmental aspects of human centricity. This could be achieved in the form of a particular case study, considering the different kinds of government structures and applicable laws, even in terms of environmental issues.

## Figures and Tables

**Table 1:** Sensor application in regard to Industry 5.0 and human-centered manufacturing.

Publication Title	Document Type	Description	Perspective of Application
Online human motion analysis in industrial context: A review [24]	Article	The article discusses the importance of human motion analysis in industrial settings, focusing on the application of various sensors like Inertial Measurement Units (IMUs), Motion Capture (MoCap) sensors, Electromyography (EMG) sensors, and Force Myography (FMG) sensors	- Safety, - Ergonomics and worker well-being, - Efficiency and productivity
From CySkin to ProxySKIN: Design, Implementation and Testing of a Multi-Modal Robotic Skin for Human–Robot Interaction [25]	Article	The paper focuses on the development of ProxySKIN, a skin-like sensory system for robots, based on networks of distributed proximity sensors and tactile sensors	- Safety, - Efficiency and productivity, - Human-robot collaboration
Infrastructure possibilities and human-centered approaches with industry 5.0 [26]	Book	This article examines the transition, emphasizing the importance of infrastructure, ethical concerns, and future implication in Human-Centric Strategies in the Context of Industry 5.0.	- Safety, - Ergonomics and worker well-being, - Human-robot collaboration
Industry 5.0: Towards Human Centered Design in Human Machine Interaction [27]	Conference paper	This paper aims to understand the human-robot interaction to achieve a complete collaboration between the robot and the human to increase efficiency, to reduce risks of possible accidents for the operator, and to prioritize their safety.	- Safety, - Efficiency and productivity, - Human-robot collaboration
Towards the Cognitive Factory in Industry 5.0: From Concept to Implementation [28]	Article	This paper provides the integration of intelligent spaces and I5.0 enables the creation of dynamic and adaptive environments that sense, interpret, recognize user behavior, and provide natural interactions between humans and intelligent systems using also by sensors.	- Efficiency and productivity, - Human-robot collaboration, - Data-driven decision making
How to provide work instructions to reduce the workers’ physical and mental workload [29]	Conference paper	The aim of the article is to redesign a workstation for wire harness assembly to mitigate ergonomic risks for operators, focusing on physical and cognitive aspects.	- Safety, - Ergonomics and worker well-being, - Efficiency and productivity
Extending factory digital Twins through human characterisation in Asset Administration Shell [30]	Article	This article is focused on extention of traditional factory digital twins by incorporating human characterisation in Asset Administration Shell (AAS) through the use of sensors.	- Safety, - Ergonomics and worker well-being
A Review of AI Cloud and Edge Sensors, Methods, and Applications for the Recognition of Emotional, Affective and Physiological States [31]	Review	This paper reviews how brain and biometric sensors can be utilized for AFFECT recognition across various domains.	- Safety, - Ergonomics and worker well-being, - Efficiency and productivity, - Human-robot collaboration, - Data-driven decision making
Hypergraph-based analysis and design of intelligent collaborative manufacturing space [32]	Article	Article explors the concept of Integrated Cyber-Physical Production Systems (ICMS) for enhancing human-machine cooperation utilizing sensors as elements of the monitoring system within the production environment.	- Efficiency and productivity, - Human-robot collaboration, - Data-driven decision making
Collaborative Robotic Environment for Educational Training in Industry 5.0 Using an Open Lab Approach [33]	Conference paper	The focus of the article is to create a safe collaborative environment for educational training in Industry 5.0 by utilizing sensor technologies such as haptics and vision systems.	- Safety, - Ergonomics and worker well-being, - Efficiency and productivity
Do We Need Synchronization of the Human and Robotics to Make Industry 5.0 a Success Story? [34]	Conference paper	This article is focused on how sensors, along with information and communications technology, connectedness, robotics, and innovative production systems, are integral to smart manufacturing systems that produce smart products	- Efficiency and productivity, - Human-robot collaboration, - Data-driven decision making

**Table 2:** The overview of relevant sources in specified fields.

Publication Title	Document Type	Short Summary	Number of Citations
Literature review of Industry 4.0 and related technologies [35]	Review	The main aim of the paper is to provide a comprehensive literature review of Industry 4.0 and related technologies to highlight progress and improve awareness for both academics and industrial practitioners.	1151
Understanding the adoption of Industry 4.0 technologies in improving environmental sustainability [36]	Article	The main aim of this paper is to study the significant benefits of industry concept for sustainable manufacturing.	164
Future of industry 5.0 in society: human-centric solutions, challenges and prospective research areas [4]	Review	This paper is focused on analysis of the potential applications of Industry 5.0, discussing definitions, advanced technologies, applications in various sectors, and challenges in human-robot interaction.	161
Socio-Technical Perspectives on Smart Working: Creating Meaningful and Sustainable Systems [37]	Article	Examination of the developments of ‘smart’ working from unique individual understandings of work roles and sustainability.	160
Link between Sustainability and Industry 4.0: Trends, Challenges and New Perspectives [38]	Review	Description of the existing relationship between industry concept and sustainability, identification of strategic themes, research gaps, challenges, and perspectives.	132
Society 5.0: A Japanese concept for a superintelligent society [39]	Review	Exploration the concept of Society 5.0 and its implications for creating a superintelligent society that addresses societal challenges through technological advancements	99
Smart Working in Industry 4.0: How digital technologies enhance manufacturing workers’ activities [40]	Article	Conceptual framework to consolidate a common view on the contribution of Industry 4.0 technologies to specific worker capabilities and manufacturing activities	87
From Supply Chain 4.0 to Supply Chain 5.0: Findings from a Systematic Literature Review and Research Directions [41]	Review	Identification of the gap related to Industry 5.0 approaches for the supply chain field and propose an alignment with the supply chain context, forming the basis for the incipient Supply Chain 5.0 framework.	68
Industry 4.0 Disruption and Its Neologisms in Major Industrial Sectors: A State of the Art [42]	Review	Highlight of the convergence of disruptive technologies like 3D printing, artificial intelligence, big data, and robotics in industries like agriculture, healthcare, and logistics	61
The emergence and rise of industry 4.0 viewed through the lens of management fashion theory [43]	Article	Explorative and qualitative research approach to sketch a comprehensive picture of the concept’s emergence and rise from inception to present day	61
An investigation upon industry 4.0 and society 5.0 within the context of sustainable development goals [44]	Article	Investigation of the Society 5.0 effectiveness and Industry 4.0 within the context of Sustainable Development Goals	60
Smart Environments and Techno-centric and Human-Centric Innovations for Industry and Society 5.0: A Quintuple Helix Innovation System View Towards Smart, Sustainable, and Inclusive Solutions [45]	Article	Investigation of the aviation sector as a case study for smart environments and Industry 5.0 and Society 5.0 purposes	54
Application of automation for in-line quality inspection, a zero-defect manufacturing approach [46]	Review	Systematic review of the literature on current trends in the application of automation for in-line quality inspection	44
Maturity assessment for Industry 5.0: A review of existing maturity models [9]	Article	Review whether the currently existing maturity models for Industry 4.0 address the specific requirements of Industry 5.0, focusing on a human-centered approach and readiness for disruptive technologies in companies, especially SMEs	41
Towards designing society 5.0 solutions: The new Quintuple Helix—Design Thinking approach to technology [47]	Article	This paper describes how to design and develop Society 5.0 (S5.0) solutions that solve social problems and benefit society by applying Industry 4.0 (I4.0) technologies.	38

**Table 3:** The list of abbreviations.

Abbreviation	Meaning
SMEs	Small and medium-sized enterprises
IR	Industrial revolution
IR 3.0	3rd Industrial revolution
AI	Artificial intelligence
IMUS	Inertial Measurement Units
MoCap	Motion Capture
EMG	Electromyography
FMG	Force Myography
ICMS	Integrated Cyber-Physical Production Systems
I5.0	Industry 5.0
I4.0	Industry 4.0
S5.0	Society 5.0
HC	Human centered
AD	Axiomatic design
CNs	Customer needs
FRs	Functional requirements
DPs	Design parameters
PVs	Process variables

## Data Availability

All data are within the paper.
